# T cell adaptation in chronic infections and tumors

**DOI:** 10.1038/s41423-026-01405-y

**Published:** 2026-03-30

**Authors:** Hendrik Luxenburger, Robert Thimme, Maike Hofmann

**Affiliations:** Department of Medicine II (Gastroenterology, Hepatology, Endocrinology and Infectious Diseases), Freiburg University Medical Center, Faculty of Medicine, University of Freiburg, Freiburg, Germany

**Keywords:** T cell exhaustion, Chronic infections, Tumors, TME, Immune checkpoint blockade (ICB), Immunological memory, Autoimmunity

## Abstract

Chronic viral infections and cancer challenge immune control by enforcing sustained antigen exposure, which profoundly alters the fate and function of CD8^+^ T cells. In contrast to acute infections, which induce robust effector differentiation and durable immune memory, persistent infections and tumors drive CD8^+^ T cells into distinct states of functional adaptation. The best studied chronic adaptation is T cell exhaustion, which is characterized by impaired effector functions, reduced proliferative capacity, sustained expression of inhibitory receptors, and stable transcriptional and epigenetic reprogramming. T cell exhaustion is not a uniform or terminal condition but comprises heterogeneous and dynamic cellular states, including stem-like/precursor populations that retain self-renewal capacity and therapeutic responsiveness. These insights have reshaped our understanding of immune regulation in chronic disease and underpin the success of immune checkpoint blockade therapies. However, heterogeneous and often transient clinical responses highlight critical gaps in our mechanistic understanding of exhausted T cell biology. This review synthesizes recent advances in the cellular and molecular profiling of chronically stimulated CD8^+^ T cells across chronic viral infection and cancer, focusing on regulatory networks, defining factors, and tissue-specific cues that govern functional adaptation and exploring emerging therapeutic reprogramming strategies.

## Introduction

Chronic viral infections and cancer represent two of the most significant global health challenges [1, 2]. While acute infections are usually resolved through a coordinated immune response that clears the pathogen and generates immunological memory, persistent infections and tumors evade or suppress immunity, leading to long-term disease [3–6]. At the center of this immunological tug-of-war are CD8^+^ T cells, the primary cytotoxic effector population of the adaptive immune system capable of recognizing and eliminating infected or malignant cells. CD8⁺ T cells play a critical role in controlling viral infections and cancer through their remarkable functional and phenotypic heterogeneity. During acute infections, antigen recognition elicits a robust and tightly coordinated T cell response marked by rapid effector differentiation, pathogen clearance, and the formation of long-lived memory populations that underpin durable protection and tumor immunosurveillance [7]. Immune memory capacity is exploited by vaccination and thus is essential for prevention [8].

In contrast, chronic infections and tumors subject CD8^+^ T cells to chronic antigen stimulation, fundamentally reshaping their effector differentiation and limiting their functional potential. Persistent TCR engagement, metabolic stress, inflammatory signals, and tissue-specific cues collectively drive an alternative differentiation pathway known as T cell exhaustion. Exhausted T (T_EX_) cells progressively lose effector functions, exhibit reduced proliferative capacity, upregulate the expression of multiple inhibitory receptors, and adopt a distinct transcriptional and epigenetic landscape [5, 6]. Rather than representing simple immune failure, exhaustion is now viewed as an adaptive state that preserves a degree of immune control while restraining excessive inflammation and immunopathology. Accordingly, chronic antigen exposure is a key driver of CD8^+^ T cell heterogeneity, influencing response dynamics and memory formation and thus the ability to control infection, prevent reinfection, and limit tumor growth.

Recognizing T_EX_ cells as a specialized differentiation program induced by chronic antigen exposure has reshaped our understanding of immunity in persistent disease and has important implications for immunotherapy. Immune checkpoint inhibitors (ICBs), such as those that block PD-1/PD-L1, CTLA-4, or LAG-3, leverage this adapted immune state by increasing the number of T_EX_ cells and have transformed cancer treatment [9]. However, variable and often transient clinical responses underscore the need for a deeper mechanistic understanding of T_EX_ biology. This review therefore integrates advances from studies of chronic infections and cancer, highlighting molecular mechanisms, cellular heterogeneity, and therapeutic strategies aimed at targeting chronically adapted T cells.

## T cell immunity in the context of acute antigen exposure

**Fig. 1:**
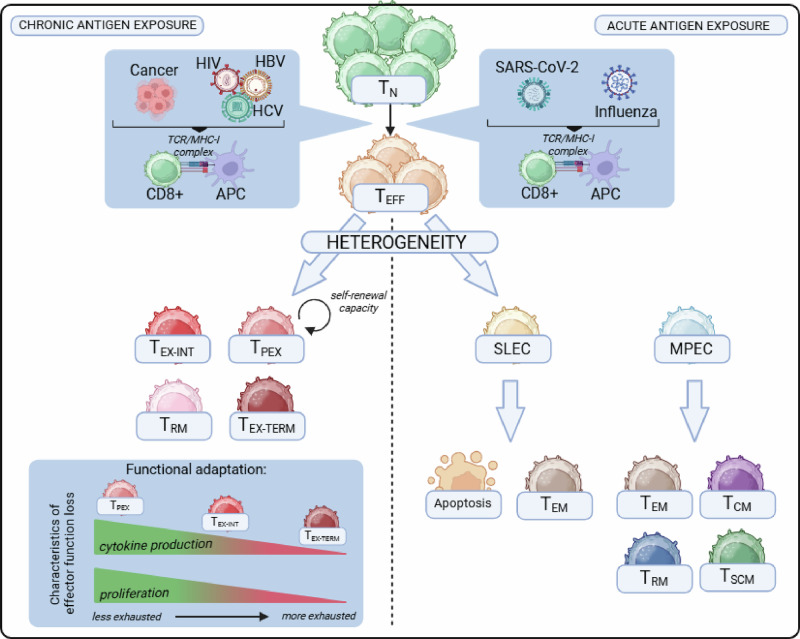
Fate of virus-specific and tumor-specific CD8^+^ T cells in the context of acute and chronic antigen exposure. Naive CD8⁺ T cells undergo priming after recognizing their cognate antigen. During acute antigen exposure, they give rise to a diverse array of effector and memory precursor subsets following activation and clonal expansion (right panel). Chronic antigen exposure leads to the redirection and adaptation of T cell differentiation and effector function toward T cells with various exhausted states, including reduced proliferation, upregulation of exhaustion markers, and epigenetic alterations (left panel). TN naive CD8^+^ T cell, T_EFF_ effector CD8^+^ T cell, SLEC short-lived effector cell, MPEC memory precursor effector cell, T_EM_ effector memory CD8^+^ T cell, T_SCM_ stem-like memory CD8^+^ T cell, TCM central memory CD8^+^ T cell, T_RM_ tissue-resident memory CD8^+^ T cell, T_PEX_ precursor exhausted CD8^+^ T cell, T_EX-INT_ intermediate exhausted CD8^+^ T cell, T_EX-TERM_ terminally exhausted CD8^+^ T cell, APC antigen-presenting cell, HIV human immunodeficiency virus, HBV hepatitis B virus, HCV hepatitis C virus, TCR T cell receptor, MHC-I major histocompatibility complex molecule class I

During acute antigen exposure, such as during a resolving viral infection, naive CD8^+^ T cells that recognize their cognate antigen undergo priming, rapid activation and clonal expansion, resulting in the formation of a diverse array of effector (short-lived effector T cells—SLECs) and memory precursor (memory precursor effector T cells—MPEC) subsets (Fig. 1). Early effector cells receive integrated signals through the TCR, costimulation (e.g., CD28), and inflammatory cytokines (e.g., type I IFN and IL-12) that drive differentiation programs characterized by cytotoxic function and the expression of transcription factors such as T-bet and RUNX3, which promote the production of granzymes, perforin, Fas ligand (FasL), and effector cytokines such as IFNγ and TNF [3, 4, 10–15]. As the infection resolves and antigens are cleared, most terminal effector cells undergo apoptosis, while a proportion of MPECs transition into long-lived memory T cells. MPECs are multipotent, with active prosurvival/memory programs and permissive effector loci, giving rise to several memory T cell subsets with the potential to express effector genes and thus enabling flexible, context-specific responses. The heterogeneous memory T cell pool comprises stem-like (T_SCM_), central (T_CM_), effector (T_EM_), and tissue-resident (T_RM_) subsets differing in proliferative, migratory, and effector characteristics (Fig. 1) [16]. T_EM_ and T_RM_ cells establish first-line sentinels in blood and peripheral tissues, whereas T_SCM_ and T_CM_ cells are highly proliferative and poised toward, but not active in, effector gene expression and are thus responsible for maintaining a highly adaptable CD8^+^ T cell memory pool [17–19]. However, emerging evidence suggests that memory T cell phenotypes exist along a continuum defined by trafficking, metabolic, and epigenetic programs rather than rigid subset boundaries, reflecting the functional flexibility and specialization required for effective host defense. Memory T cells can persist and mount rapid and robust responses upon re-exposure to the same pathogen. Hence, this heterogeneity and plasticity in acute responses ensure both effective pathogen clearance and durable immunological memory.

## T cell immunity in the context of chronic antigen exposure

Persisting infections, e.g., those involving human immunodeficiency virus (HIV), hepatitis B virus (HBV), and hepatitis C virus (HCV), as well as tumors, represent major health burdens [1, 2]. T cells are key determinants of the outcome and pathogenesis of these diseases and represent promising therapeutic targets, e.g., in ICB therapy. Chronic antigen exposure and stimulation associated with persistent infections and tumors drive the redirection and adaptation of T cell differentiation and effector function toward restrained and exhausted states rather than toward durable effector or memory fates. Concomitantly, suppressive networks, e.g., those involving regulatory T cells, myeloid-derived suppressor cells (MDSCs), inhibitory cytokines, and metabolic checkpoint pathways, reinforce this skewing of T cell differentiation [20–22]. While T cell adaptation to chronic stimulation favors viral persistence and tumor progression, it also limits overwhelming immunopathology and ensures the maintenance of residual T cell immunity, partly controlling viral replication and restraining tumor growth [23]. The physiological relevance of T cell adaptation to persisting stimulation is supported by the fact that T cell populations capable of ameliorating chronic antigen exposure are preemptively formed during the acute phase, enabling the immune system to adapt to differential outcomes of immune threats [24–26]. Notably, T cell adaptation programs appear to be reversible only in part, and epigenetic constraints limit full reprogramming. Understanding these constraints is central to improving immunotherapies targeting chronic infection and cancer.

### Definition of T cell exhaustion

T cell adaptation to chronic antigen exposure is best studied for CD8^+^ T cells and is commonly referred to as T cell exhaustion. T cell exhaustion was initially described as a progressive state of T cell dysfunction in chronic lymphocytic choriomeningitis virus (LCMV) infection [27, 28], characterized by a gradual loss of effector functions such as cytokine production and proliferation under persistent antigen stimulation and inflammation, ultimately leading to terminal dysfunction (Fig. 1). Moreover, T cell exhaustion is recognized as a distinct differentiation state or lineage of T cells rather than transient or incidental dysfunction, resulting in distinct functional and phenotypic features, altered metabolism, and a unique transcriptional and epigenetic landscape [5, 29–33]. T cell exhaustion differs from T cell anergy and senescence: anergy is a hyporesponsive state triggered by antigen encounter without adequate costimulation, leading to functional unresponsiveness without major lineage commitment [34, 35], whereas senescence reflects replicative or stress-induced cell aging, characterized by shortened telomeres, altered metabolism, and limited proliferative capacity [36]. Since its initial description, T cell exhaustion has been observed in multiple chronic infections, including hepatitis C virus (HCV), hepatitis B virus (HBV), and human immunodeficiency virus (HIV), as well as in various cancers in both mice and humans, highlighting the translational relevance for the mechanistic understanding of a broad range of diseases with persistent immune activation.

### Phenotypic, functional, and metabolic features of T cell exhaustion

**Fig. 2:**
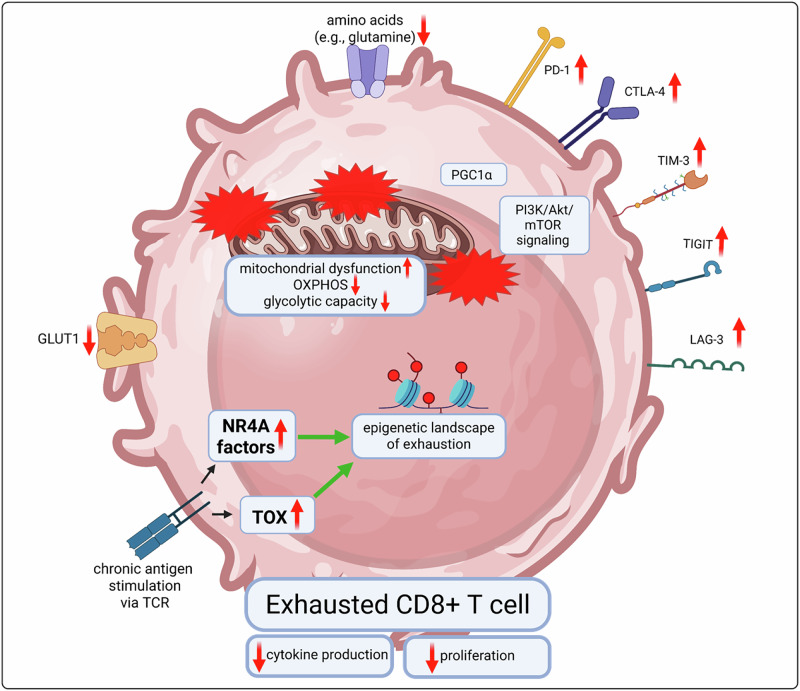
Characteristics of T cell exhaustion. Exhausted T cells (T_EX_) are characterized by a complex interplay of metabolic impairment, reduced functionality, epigenetic alterations, and the upregulation of inhibitory receptors. GLUT1 Glucose transporter 1, TOX Thymocyte selection-associated high mobility group box protein, NR4A nuclear receptor subfamily 4A, OXPHOS Oxidative phosphorylation, PGC-1α peroxisome proliferator-activated receptor gamma coactivator 1-alpha, TCR T cell receptor

T_EX_ cells are characterized by the coordinated and sustained upregulation of the expression of multiple inhibitory receptors, including PD-1, LAG-3, TIM-3, TIGIT, and CTLA-4, which collectively decrease T cell receptor (TCR)-mediated activation and limit effector functions [5, 22]. Although an overarching principle of these inhibitory receptors is the recruitment of phosphatases such as Src homology region 2 domain-containing phosphatase (SHP)-2 or SHP-1, they also act slightly differently [37]. Indeed, PD-1 primarily acts directly on the TCR/CD28 signaling cascade via phosphatase recruitment, while CTLA-4 mainly blocks costimulatory signals by outcompeting CD28 [38, 39]. Thus, these inhibitory receptors serve not only as markers but also as functional regulators, and their expression is accompanied by a progressive loss of effector cytokine production, most notably that of IL-2, TNF, and, ultimately, IFNγ, reflecting a stepwise decline in functional competence. In parallel, T_EX_ cells exhibit reduced cytotoxic activity and impaired proliferative capacity, limiting their ability to clear infected or malignant cells effectively. Notably, the different inhibitory receptors play nonredundant roles in regulating T cell effector functions. For example, PD-1 is linked to the diminished proliferative capacity of T_EX_ cells and LAG-3 to restrain cytokine production and cytotoxicity and maintain the transcriptional T_EX_ program [40–42]. Deficits in functional T_EX_ cells are underpinned by profound metabolic dysregulation, including mitochondrial dysfunction, reduced oxidative phosphorylation (OXPHOS), and restricted glycolytic capacity, which further constrain the energy availability and biosynthetic support required for proliferation and effector responses [5, 6, 41] (Fig. 2). These metabolic alterations in T_EX_ cells are also partly mediated by inhibitory receptors. For example, PD-1 profoundly modulates the metabolism of T_EX_ cells by directly interfering with key signaling pathways that regulate energy production and biosynthesis. Inhibiting PI3K/Akt/mTOR signaling results in reduced glycolytic flux, limiting the rapid energy production needed for proliferation and effector function [43]. PD-1 signaling also suppresses peroxisome proliferator-activated receptor gamma coactivator 1-alpha (PGC-1α), reducing mitochondrial mass and membrane potential and reducing respiratory capacity and thus impairing OXPHOS and ATP generation [43]. The combination of glycolytic and mitochondrial restriction creates a metabolically starved cell state. In addition, PD-1 signaling reduces the uptake and utilization of amino acids (e.g., glutamine) and fatty acids, further constraining the anabolic pathways needed for effector functions [44]. T_EX_ cells therefore shift toward a catabolic, low-energy metabolic program. In addition to inhibitory receptors, T_EX_ cells also express the enzymatically active ectonucleotidase CD39, which plays a critical role in regulating the metabolic and functional state of T_EX_ cells [45]. It hydrolyzes extracellular ATP and ADP into AMP, which is further converted to adenosine by CD73, which is expressed by multiple immune and nonimmune cells, including T_EX_ cells themselves. Adenosine acts immunosuppressively via adenosine receptors on T cells to increase intracellular cAMP levels, leading to the inhibition of TCR signaling and subsequently reducing cytokine production and proliferation [46]. This depletion of extracellular ATP by CD39 also suppressed mTOR-driven metabolic pathways, leading to restricted glycolysis, mitochondrial dysfunction, and reduced energy availability, and strengthening the T_EX_ cell state. Notably, metabolic reprogramming also stabilized the transcriptional and epigenetic programs of T_EX_ cells. Together, these features define a metabolically and functionally restrained state, which is distinct from acute activation or senescence and represents a hallmark of chronic antigen exposure and persistent immune stress.

### Transcriptional and epigenetic programs of T cell exhaustion

T_EX_ cells are based on a distinct transcriptional program driven by a network of so-called lineage-defining transcription factors. Key regulators include TOX (Thymocyte selection-associated high mobility group box protein), which imposes an epigenetic landscape that restricts effector differentiation, and NR4A family members, which dampen TCR signaling. This transcriptional network is strongly connected to epigenetic remodeling, enforcing the stability of the T_EX_ program and setting limits on T_EX_ plasticity. ATAC-seq and single-cell chromatin analyses revealed that T_EX_ cells acquire enhancer landscapes distinct from those of both effector and memory T cells, with exhaustion-specific open chromatin regions enriched for TOX and NR4A motifs [47–52].

TOX expression is induced by persistent TCR stimulation and NFAT signaling, marking the transition from effector to exhausted states. TOX induces the upregulation of inhibitory receptors while repressing the expression of effector cytokine genes such as *Il-2*, *Tnf*, and *Ifnγ*. Functionally, TOX helps stabilize the epigenetic landscape of exhaustion, including chromatin accessibility at loci associated with inhibitory receptors and metabolic regulators, thereby creating a durable exhausted phenotype that is resistant to full functional reinvigoration [47–50]. TOX contributes to metabolic adaptation in T_EX_ cells. In particular, TOX downregulates the expression of glycolytic genes such as *Glut1* (*Slc2a1*), indicating a low-glycolysis, energy-conserving state. TOX also modulates amino acid metabolism by partially suppressing the expression of transporters such as *Slc7a5* (*LAT1*) and by repressing the expression of genes related to glutamine metabolism, which in turn favors catabolic programs over anabolic growth. Together, these coordinated transcriptional effects position TOX as a key regulator that reprograms T_EX_ metabolism to maintain minimal energy production, promote survival, and sustain long-term persistence in metabolically restrictive microenvironments [52, 53]. In summary, TOX is considered a lineage-defining factor of T_EX_ cells that integrates chronic antigen signaling, inhibitory receptor expression, epigenetic remodeling, and metabolic reprogramming to maintain the exhausted state (Fig. 2).

The nuclear receptor subfamily 4A (NR4A) family of transcription factors (NR4A1/Nur77, NR4A2/Nurr1, and NR4A3/Nor1) is induced in T_EX_ cells primarily through persistent TCR signaling during chronic antigen exposure. Sustained TCR engagement activates the calcineurin–NFAT pathway, and in the context of chronic stimulation, NFAT is translocated to the nucleus largely without AP-1, directly driving the transcription of *Nr4a* genes [51, 54]. In contrast, during acute infection, NFAT acts together with AP-1. Additionally, compared with acutely activated effector T cells, T_EX_ cells exhibit sustained expression of NR4A factors. Once expressed, NR4A directly and indirectly promotes the transcription of multiple inhibitory receptor genes, including *Pdcd1* (encoding PD-1), *Havcr2* (encoding TIM-3), *Lag3*, *Ctla*4, and *Tigit*, stabilizes exhaustion-specific epigenetic landscapes, and coordinates metabolic adaptation [52]. NR4A factors, therefore, link chronic antigen stimulation to the establishment and maintenance of the T_EX_ phenotype. These genes mostly act in coordination with other transcriptional regulators, such as TOX [52]. NR4A factors also transcriptionally regulate *Bcl2* family genes, balancing proapoptotic (e.g., Bim) and antiapoptotic (e.g., Bcl-2, Bcl-xL) signals and decreasing the balance toward survival in T_EX_ cells. Thus, NR4A helps to limit activation-induced cell death (AICD) by directly interfering with the apoptotic machinery in addition to supporting the T_EX_ phenotype, further supporting long-term survival despite chronic antigen exposure [51]. Hence, NR4A family members act as lineage-stabilizing transcription factors in T cell exhaustion, linking chronic TCR stimulation to sustained inhibitory receptor expression, epigenetic remodeling, metabolic adaptation, and long-term survival (Fig. 2).

Importantly, transcriptional and epigenetic T_EX_ states persist even after the removal of antigen or therapeutic intervention, which explains why exhausted T cells rarely convert into bona fide effector/memory T cells despite functional reinvigoration after ICB treatment [53, 55–58]. Targeting epigenetic regulators such as histone-modifying enzymes or chromatin remodelers offers a strategy to reshape these programs [59], although careful titration and a more detailed understanding of the transcriptional/epigenetic network are needed to avoid promoting immunopathology by interfering with the adapted T_EX_ state.

### T_EX_ cell heterogeneity

Characteristics associated with T cell exhaustion are not uniform across the T_EX_ cell compartment. Instead, T_EX_ cells constitute a heterogeneous population reflecting a differentiation continuum with functional specialization [60–63] (Fig. 1). Different T_EX_ subsets can be distinguished on the basis of their transcriptional, phenotypic, functional, and spatial characteristics. Among these, a key distinction exists between precursor T_EX_ cells (T_PEX_) and terminal T_EX_ cells, a dichotomy that has become a foundational concept for modern immunotherapy design. T_PEX_ cells are characterized by the expression of TCF-1 and intermediate levels of PD-1 and a more permissive chromatin configuration. These cells are typically localized in lymphoid tissues, where they are maintained by supportive microenvironments and can give rise to exhausted downstream subsets. TCF-1 (T cell factor 1, encoded by *Tcf7*) is a lineage-associated transcription factor in T_EX_ cells that promotes the expression of genes such as *IL7RA* (CD127), which is associated with proliferative capacity, self-renewal, survival, and metabolic flexibility, while restraining terminal exhaustion programs by limiting TOX, Eomes, and other transcriptional drivers of terminal differentiation. Among TCF-1^+^ T_PEX_ cells, a CD62L-expressing subset is particularly responsible for long-term proliferative potential, multipotency, and repopulation capacity, thus representing stem-like T_EX_ cells. In concert with TCF-1, the transcription factor MYB plays a crucial role in the development of these CD62L^+^ stem-like T_EX_ cells and regulates genes associated with the cell cycle and survival [64]. Notably, MYB also induces functional exhaustion and therefore supports the orchestration of both aspects of T cell exhaustion, a reduction in immunopathology, and the preservation of T cell immunity. Overall, TCF-1 defines a stem-like/precursor hierarchy within the T_EX_ cell pool, integrating transcriptional, epigenetic, and metabolic programs that sustain long-lived T_EX_ cell populations [65]. Importantly, TCF-1^+^ T_PEX_ cells act as the primary responders to PD-1 blockade, as their proliferative capacity and retained functional plasticity allow them to expand and differentiate into more cytotoxic progeny upon checkpoint inhibition [66, 67]. However, despite this knowledge that TCF-1^+^ T_PEX_ cells are key targets of checkpoint inhibition, we still do not have biomarkers for predicting differential therapeutic responses at the individual patient level or for different tumor entities and chronic infections. Mechanisms underlying this differential response to checkpoint inhibition probably include further TCF-1^+^ T_PEX_ heterogeneity and the ability of TCF-1^+^ T_PEX_ cells to act within certain microenvironments.

In contrast, terminal T_EX_ cells lack TCF-1 expression (TCF-1⁻), express high levels of PD-1 and TIM-3, and exhibit severely impaired proliferative potential. Terminal differentiation of T_EX_ cells is enforced by lineage-associated Eomes. In particular, Eomes acts via a distinct cooperation network of Eomes and T-bet, with the characteristic expression pattern Eomes^hi^/T-bet^dim^ contributing to the loss of effector cytokine production and low-level expression of cytotoxic molecules uncoupled from an effective cytotoxic response [68]. Additionally, it acts downstream and is supportive of TOX. Moreover, in terminal T_EX_ cells, the transcriptional repressor Blimp-1 antagonizes the expression of memory- and self-renewal genes (e.g., *Tcf7* encoding TCF-1, IL7R*IL7R*, and *Bcl*6), promoting inhibitory receptor expression and limiting proliferation and survival by interfering with CD25, MYC, and Bcl-2 expression [69]. Terminal T_EX_ cells are usually enriched in peripheral tissues, including chronically infected tissues and tumor sites, where they display a locked epigenetic profile and subsequent minimal responsiveness to immunotherapeutic interventions. The distinction between precursor/stem-like and terminal T_EX_ cells in terms of response to checkpoint inhibition underscores the importance of a more detailed understanding of T_EX_ cell heterogeneity for therapeutic design.

PD-1 plus IL-2 “cis” combination therapy, for example, revealed an additional T_EX_ subset often referred to as effector-like cells [70, 71]. Effector-like T_EX_ cells are characterized by the expression of natural killer (NK) cell receptors, which increase their cytotoxic potential, and their differentiation is influenced by IL-2 signaling, which partly overcomes the limitations of proliferation and effector function despite chronic antigen exposure [72]. These cells occupy an intermediate functional niche between proliferative stem-like/precursor T_EX_ subsets and the terminally exhausted population, contributing to local cytotoxic responses while remaining constrained by inhibitory receptor-mediated regulation. Importantly, effector-like T_EX_ cells demonstrate the plasticity of the exhaustion program, emphasizing that exhausted cells are not in a terminal state but rather in a dynamic continuum of functional capacities shaped by transcription factors, the cytokine milieu, and tissue localization. BATF is considered to regulate the balance between effector differentiation, progenitor maintenance, and the terminal exhausted state during chronic antigen exposure [73, 74]. It acts as a lineage-supporting transcription factor in T cell exhaustion in concert with TOX and NR4A. BATF maintains progenitor T_EX_ cell populations and guides their gradual transition toward terminal T_EX_ cells. Current evidence clearly indicates that the transcriptional and epigenetic regulatory networks underlying T cell exhaustion are governed by a finely tuned balance among lineage-determining, lineage-supporting, and lineage-associated transcription factors, which ultimately dictates whether a T_EX_ cell preserves its proliferative potential or commits to a terminal state.

Overall, the heterogeneity of T_EX_ cells, encompassing stem-like/precursor, effector-like, and terminal subsets, highlights a hierarchical structure that integrates transcriptional and epigenetic programming, tissue distribution and residency, proliferative potential, and responsiveness to therapeutic intervention. Stem-like/precursor T_EX_ cells act as self-renewing reservoirs that generate downstream populations in effector-like and terminal T_EX_ states. This heterogeneity is critical for immunotherapy, as effective strategies such as checkpoint blockade, adoptive T cell transfer, and combination therapies must preferentially target and expand the stem-like compartment while acknowledging the limited plasticity and imprinted adaptation to achieve durable immunity while still limiting immunopathology.

## T cell immunity in chronic viral infections: lessons from humans

Chronic infections with HIV, HCV, and HBV remain major global health burdens, affecting hundreds of millions of people worldwide and leading to long-term morbidity and mortality. These viruses establish persistent infections by evading or subverting host immune responses, resulting in ongoing viral replication, chronic inflammation, and progressive tissue damage. Adaptation of the T cell response to continuous antigenic stimulation is evident in these clinically relevant infections and represents a central determinant of disease pathogenesis and clinical outcome. Although antiviral therapies have markedly improved the control of viral replication, chronic HIV, HCV, and HBV infections continue to pose significant challenges because of incomplete immune restoration, viral persistence, and the risk of long-term complications.

A more detailed mechanistic understanding of immune alterations, particularly those affecting T cell immunity, is therefore essential to optimize and sustain therapeutic strategies. Recent advances have highlighted that the heterogeneity and dynamic nature of T cell responses in human chronic infections both expand upon and diverge from mechanisms described in mouse models, with distinct features emerging across different infections. These disease-specific patterns underscore the critical influence of microenvironmental factors, viral tropism, and antigen load on T cell fate and function, ultimately shaping clinical manifestations and outcomes. The characteristics of CD8⁺ T cell immunity in chronic HIV, HCV, and HBV infection are summarized in Table 1.

**Table 1:** Characteristics of CD8⁺ T cell immunity in chronic HIV, HCV, and HBV infection

	Common characteristics of virus-specific CD8^+^ T cell response in chronic infection	Distinct findings of virus-specific CD8^+^ T cell response in chronic infection	References
HIV	Main mechanisms of CD8^+^ T cell adaptation include: 1. T cell exhaustion with: - Expression of inhibitory receptors, such as LAG-3, TIM-3, TIGIT, 2B4, and CD160 - Impaired effector function - Reduced frequencies of virus-specific CD8^+^ T cells - Altered metabolism - Distinct transcriptional and epigenetic programs 2 Viral escape leading to an impaired antigen recognition	Early initiation of HIV therapy can preserve a certain degree of multifunctionality of HIV-specific CD8^+^ T cells Regulatory T cells promote T cell exhaustion through IL-10–mediated suppression of T cell proliferation	77, 78, 81
HCV		Heterogeneous T_EX_ subsets, such as the memory-like TCF-1 + CD127 + PD-1+ and terminally differentiated Eomes^high^TOX^high^CD127-PD-1+ subsets TCF-1+ memory-like T cells mount recall responses after successful DAA therapy, but remain functionally restrained compared with classical memory CD8⁺ T cells Differences between memory-like and bona fide memory CD8^+^ T cells are caused by epigenetic imprinting and transcriptional scarring Reduced efficacy despite measurable T cell responses in HCV vaccine trials, likely due to viral escape	29, 48, 55–57, 63, 86–89
HBV		Inhibitory molecules are variably expressed, but no correlation with functional impairment HBV-specific CD8⁺ T cell responses differ depending on antigen specificity Liver rheostat, lack of costimulation, and TGF-β-associated T cell attenuation are additional mechanisms of the adapted HBV-specific CD8^+^ T cell response	93–96, 105

*HIV* human immunodeficiency virus, *HCV* hepatitis C virus, *HBV* hepatitis B virus, *T*_EX_ exhausted T cells, *DAA* direct-acting antiviral, *CREM* cAMP-responsive element modulator, *TCR* T cell receptor

### CD8^+^ T cell immunity in HIV infection

HIV primarily targets the immune system itself, leading to immune dysfunction and acquired immunodeficiency. Chronic HIV infection is characterized by persistent viral replication, profound CD4^+^ T cell depletion, and a progressive loss of immune control [75–77]. Sustained antigen exposure drives HIV-specific CD8^+^ T cells into an exhausted state, impairing their cytotoxic function and ability to eliminate infected cells. Factors contributing to T cell exhaustion include persistent viral antigens, chronic proinflammatory immune activation, and disrupted T cell homeostasis. Notably, early initiation of HIV therapy to suppress viral replication can preserve a certain degree of the multifunctionality of HIV-specific CD8^+^ T cells [78].

The exhausted state of HIV-specific CD8^+^ T cells largely reflects features identified in LCMV-specific CD8^+^ T cells during chronic infection. Longitudinal studies performed in HIV progressors and rare HIV controllers have indicated that increasing PD-1 expression on HIV-specific CD8⁺ T cells precedes the loss of viral control, highlighting the fine balance between effective T cell immunity and progressive exhaustion [77]. While PD-1 is primarily correlated with viral load and disease progression, HIV-specific CD8^+^ T cells are characterized by the expression of other inhibitory receptors, including LAG-3, TIM-3, TIGIT, 2B4, and CD160, that regulate restrained effector function [77]. Thus, the effector functions of HIV-specific CD8^+^ T cells can be partially enhanced by checkpoint blockade. The effector cell capacities of HIV-specific CD8^+^ T cells are also limited by CD39, which is especially associated with terminal functional exhaustion [45]. Furthermore, HIV-specific CD8⁺ T cells display increased susceptibility to apoptosis, which is associated with reduced Bcl-2 expression and elevated CD95/Fas expression. The differentiation program of exhausted HIV-specific CD8^+^ T cells is guided by high Eomes expression and low T-bet expression and is fixed by epigenetic remodeling, e.g., by reduced chromatin accessibility at the IFNγ locus [79, 80].

Immunosuppressive cytokines such as IL-10 also contribute to the exhausted T cell state during HIV infection, which is driven in part by sustained activation of type I and II interferon pathways [77]. Regulatory T cells also accumulate and directly promote T cell exhaustion through the IL-10–mediated suppression of T cell proliferation [81]. Along these lines, elevated plasma IL-10 levels are associated with rapid disease progression and impaired CD4⁺ T cell help, whereas in vitro blockade of IL-10 enhances the proliferation of HIV-specific T cells [77]. All these phenotypic and mechanistic determinants culminate in a wide spectrum of exhausted virus-specific CD8^+^ T cells comprising core subsets shared with the mouse LCMV model as well as disease-specific subsets uniquely associated with persistent HIV infection [31]. The composition and diversification of these subsets correlate with disease severity and changes in response to HIV therapy, highlighting exhausted T cell populations as potential sentinels for immune monitoring in humans. Overall, HIV-specific T cell exhaustion, which is established early after infection, constitutes a major barrier to immune-mediated viral control and clearance of infected cells and closely mirrors the clinical course of disease progression and response to treatment.

### CD8^+^ T cell immunity in chronic HCV infection

HCV predominantly infects the liver, where chronic infection can drive fibrosis, cirrhosis, and hepatocellular carcinoma. Unlike other chronic viral infections, such as HIV or HBV, for which long-term antiviral therapy rarely achieves viral clearance, the introduction of direct-acting antivirals (DAAs) has revolutionized HCV treatment, resulting in cure rates exceeding 90% across all genotypes [82, 83]. This makes HCV infection a unique immunological model for studying the plasticity of immunity established during and after chronic infection. Infection with HCV induces a strong type I IFN response and elicits robust virus-specific CD8^+^ T cell immunity [84, 85]. In chronic HCV infection, however, the frequency of virus-specific CD8^+^ T cells is reduced, and these cells are functionally impaired and fail to eliminate the virus, subsequently contributing to ongoing liver disease [86]. Persisting HCV replication is accompanied by HCV-specific CD8^+^ T cell exhaustion, which is already early during the course of chronic infection [87]. In line with the classical differentiation program of T cell exhaustion established in a mouse model of chronic LCMV infection, exhausted HCV-specific CD8^+^ T cells are characterized by the increased expression of inhibitory receptors (e.g., PD-1, TIM-3, LAG-3, and 2B4), altered metabolism and distinct transcriptional and epigenetic programs, including high Eomes and TOX activity but low T-bet expression [29, 48, 55–57, 63, 88]. Exhausted HCV-specific CD8^+^ T cells are heterogeneous and include memory-like TCF-1^+^CD127^+^PD-1^+^ subsets, corresponding to the stem-like/precursor subsets described in LCMV infection, and terminally differentiated Eomes^high^TOX^high^CD127^-^PD-1^+^ subsets [55, 89]. TCF-1^+^ HCV-specific CD8⁺ T cell subsets display enhanced proliferative capacity, can differentiate into terminally exhausted cells, and can persist independent of antigens. This is particularly notable because, following curative DAA therapy for chronic HCV infection, the TCF-1^+^ subset established during chronic infection forms a durable T cell memory, which is why they are termed memory-like cells [89]. Remarkably, these memory-like cells can mount recall responses and persist for years, exhibiting key features of immune memory. Nevertheless, they remain functionally restrained compared with classical memory CD8⁺ T cells generated after spontaneous HCV clearance, highlighting the epigenetic imprinting and transcriptional scarring imposed by chronic infection [55].

**Fig. 3:**
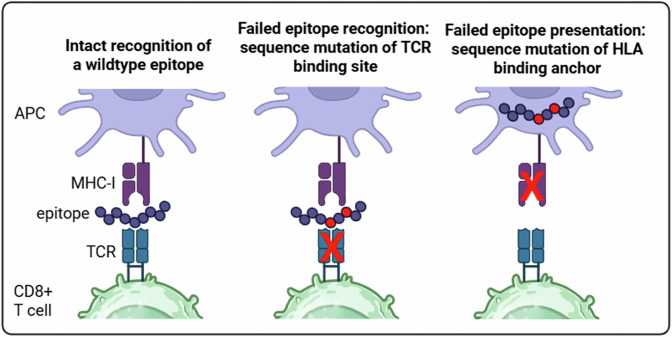
Mechanisms of viral escape. Viral escape may occur within HLA-binding anchors or TCR-binding sites, leading to failed epitope presentation and recognition. APC antigen-presenting cell, TCR T cell receptor, MHC-I major histocompatibility complex molecule class I

In chronic HCV infection, viral clearance failure is also frequently driven by viral escape mutations that reduce or even abrogate antigen recognition by T cells (Fig. 3). More precisely, sequence polymorphisms in the viral genome can occur in or near viral epitopes, thereby hindering their recognition by epitope-specific T cells, consequently called escape mutations [90]. While viral escape from T cells also occurs in HIV infection, it is particularly frequent in HCV because of the single-stranded RNA genome and its error-prone replication at high rates. In HCV infection, escape mutations often occur in immunodominant CD8^+^ T cell epitopes or their flanking regions, impairing antigen recognition or presentation and thus T cell activation, and are more common in CD8^+^ than in CD4^+^ epitopes. Escape mutations limit persistent antigen stimulation, thereby affecting CD8^+^ T cell exhaustion dynamics. In particular, virus-specific CD8⁺ T cells targeting escaped epitopes adopt a predominantly TCF-1^+^ phenotype, whereas T cells recognizing conserved epitopes show TCF-1^+^ and terminally exhausted subsets [89]. Additionally, the transcriptome of virus-specific CD8^+^ T cells targeting escaped epitopes shows a mixed regulatory pattern combining programs of T cell effector/memory differentiation and exhaustion [55, 57]. These observations support the role of antigen recognition dynamics in the T cell exhaustion program. The widespread occurrence of viral escape at both the individual and population levels has significant consequences for vaccine development, as illustrated by HCV vaccine trials in which escape mutations likely reduced protective efficacy despite measurable T cell responses. Overall, viral escape, in combination with T cell exhaustion, represents a key mechanism underlying immune control, therapeutic success, and vaccine effectiveness in chronic infections.

### CD8^+^ T cell immunity in chronic HBV infection

Like HCV, HBV is a noncytopathic virus with strict tropism for human hepatocytes. In contrast to HCV, HBV elicits only weak activation of the innate immune system, most likely resulting in delayed emergence of a T cell response [91]. In addition, high levels of subviral particles, which consist of viral envelope proteins (HBsAg), are subsequently released into the circulation, resulting in high antigen load. Although HBV infection can lead to fibrosis, cirrhosis, and hepatocellular carcinoma, the human immune system is often capable of controlling viral replication, albeit it rarely achieves complete viral elimination.

Virus-specific CD8⁺ T cells play a pivotal role in determining the outcome of HBV infection, and chronic HBV infection is characterized by profound quantitative and functional T cell impairments [92]. Notably, dysfunction of HBV-specific CD8^+^ T cells in chronic infection does not uniformly match the classical paradigm of T cell exhaustion [93]. Although these cells are present at low frequencies in chronically infected individuals, they display considerable heterogeneity in both inhibitory receptor expression and effector capacity. Inhibitory molecules such as PD-1, TIM-3, CTLA-4, and CD160 are variably expressed, yet their presence does not consistently correlate with functional impairment [94]. These observations suggest that noncanonical mechanisms contribute to the regulation and adaptation of HBV-specific CD8^+^ T cell responses during chronic infection.

HBV-specific CD8⁺ T cell responses differ depending on antigen specificity [95, 96]. Envelope/HBsAg-specific CD8⁺ T cells are frequently undetectable in chronically infected adults, probably because of the high antigen load, whereas T cells targeting core and polymerase antigens are more common and retain varying degrees of functionality. Compared with polymerase-specific CD8^+^ T cells, core-specific CD8^+^ T cells generally display higher frequencies and superior proliferative capacity. These functional differences are accompanied by distinct transcription factor expression patterns, including those of TOX and metabolic markers such as enolase, further emphasizing the heterogeneity of the HBV-specific CD8^+^ T cell compartment in the context of persistent antigen exposure [97, 98].

The mechanisms underlying this pattern of dysfunction are multifactorial and differ from those driving conventional T cell exhaustion. In addition to the established influence of liver fibrosis and metabolic constraints such as arginine deprivation on CD8^+^ T cell function within the liver, several additional factors have been identified [92, 99–101]. Impaired T cell priming in the context of limited innate immune activation and an atypical priming environment in which hepatocytes themselves can directly activate CD8^+^ T cells [102]. This process induces a nonclassical transcriptional program characterized by partial effector function coupled with restrained differentiation, resembling tolerance or anergy rather than classical exhaustion. Such cells respond poorly to checkpoint blockade but can be more effectively reactivated by costimulation, such as with IL-2 or 4-1BB agonists [103, 104]. Additionally, the liver microenvironment imposes further regulatory constraints on liver sinusoidal endothelial cells (LSECs), which directly interact with intrahepatic CD8^+^ T cells [105]. During persistent hepatic infection, the resulting menage a trois, formed by hepatocytes, CD8^+^ T cells, and LSECs, enhances adenylyl cyclase-cAMP-PKA signaling, increases cAMP-responsive element modulator (CREM) activity, and impairs TCR signaling, thereby restraining T cell activation and effector function in an immune rheostat–like manner [105]. Moreover, cytokines can shape CD8^+^ T cell immunity during chronic HBV infection. For example, TGF-β has been shown to shape a subset of polymerase-specific CD8⁺ T cells that, despite reduced effector differentiation, retain potent cytotoxic potential [106]. Through the downregulation of transcription factors such as ZEB2 and T-bet, cytokine modulation by TGF-β promotes an attenuated effector cell state, which challenges the notion that HBV-specific CD8⁺ T cells are uniformly exhausted. This observation highlights therapeutic opportunities for increasing the number of virus-specific CD8^+^ T cells through targeted pathway modulation, particularly since attenuated polymerase-specific CD8^+^ T cells have been closely linked to the clinical stage of endogenous viral control in chronic HBV infection without the need for current treatment.

Collectively, chronic HBV infection is associated with remarkable diversity among HBV-specific CD8^+^ T cell populations, reflecting the complex interplay between viral strategies of immune evasion and host regulatory mechanisms and underscoring the need for a nuanced understanding of CD8^+^ T cell adaptation in chronic infection. This finding further supports the rationale for immunotherapeutic approaches tailored to specific immune profiles and most likely has implications for T cell adaptation to chronic stimulation beyond infection, namely, tumors.

### T cell dysfunction in tumors

T cell dysfunction in chronic infections and tumors has several similarities, including progressive loss of effector function, upregulation of inhibitory receptors (e.g., PD-1 and LAG-3), and altered transcriptional and epigenetic landscapes [5, 6]. However, several key mechanistic and contextual differences exist. While persistent antigen stimulation and proinflammatory cytokines are the main drivers of T cell differentiation in chronic infection, in tumors, a dynamic interplay between the host immune system and the tumor accounts for T cell dysfunction. This process, known as cancer immunoediting, involves the immune system controlling and shaping cancer. It occurs through three phases: elimination, equilibrium, and escape [107, 108]. The elimination phase is defined by the interaction between the innate and adaptive immune systems, which aims to eradicate highly immunogenic tumor cells by inducing direct cytotoxicity, apoptosis, and phagocytosis [109]. However, reduced immunogenicity or acquired resistance can lead to the survival of tumor cells and the transition to the equilibrium phase. During this phase, the adaptive antitumoral immune response exerts selective pressure on tumor cells, which is mediated primarily by T cells. This leads to the immunoselection of less immunogenic variants. These tumor cells exist in a state of dynamic equilibrium, in which they are not eradicated, but their growth is restrained by ongoing immune surveillance [110]. Finally, during the escape phase, tumors grow progressively and establish immunosuppressive niches that enable chronic antigen stimulation and T cell dysfunction, causing clinically apparent disease [110, 111]. These immunosuppressive niches, also known as the tumor microenvironment (TME), are composed of different components, including stromal cells, endothelial cells, fibroblasts, metabolic gradients, and infiltrating immune cells [112]. In the TME, T cells are constantly exposed to tumor antigens, inhibitory ligands, suppressive cytokines, and metabolic stress, causing phenotypic alterations [113]. However, these tumor-infiltrating T cells (TILs) are not uniform. Over the past decade, single-cell transcriptomics, epigenetic analyses, and TCR sequencing have revealed that these cells are present in diverse states ranging from effective cytotoxic cells to early and severely exhausted dysfunctional T cells [113]. Understanding these states and the pathways that govern them is central to designing next-generation immunotherapies.

### TIL heterogeneity

TILs are composed of different cell populations, including CD8^+^ and CD4^+^ T cells (including regulatory T cells) and innate-like T cells. TILs can either act independently of the tumor or be tumor reactive [113]. The tumor reactivity of TILs is driven by a variety of tumor antigens, which are defined as molecules expressed by cancer cells that enable immune recognition. Tumor antigens are broadly classified by their origin and expression pattern. They include tumor-associated antigens (TAAs) or tumor-specific neoantigens [114, 115], which arise from somatic mutations unique to tumors. TAAs are common and cover differentiation antigens linked to tissue lineages, proteins that are overexpressed or amplified in cancer, and cancer-testis antigens that are normally restricted to germ cells [116]. Additional classes include aberrantly glycosylated mucins and glycoproteins, lineage-restricted surface markers characteristic of hematologic malignancies, and oncofetal antigens re-expressed during tumorigenesis [117]. Virus-associated cancers present viral antigens, while metabolic and stress-induced proteins are also frequently upregulated in response to malignant transformation [115]. Notably, alterations in antigen presentation can be frequently observed as a tumor evasion strategy. Diminished antigen presentation may result from defects in antigen processing and presentation, including HLA-I/II loss of heterozygosity and loss of functional β2-microglobulin [118]. Driven by immune selection pressure, some tumors also selectively lose the expression of dominant neoantigens targeted by tumor-specific T cells, resulting in antigen escape [119]. This variety of tumor antigen quality and presentation, as well as spatiotemporal cues within tissues and lymphoid sites, determines heterogeneous T cell fates and subsequent responsiveness to immunotherapies.

Heterogeneous TIL fates can consequently be grouped by their mode of action and migratory behaviors. TILs include recently recruited effector T cells or exhausted T cells that retain the capacity to recirculate. Recently, recruited cytotoxic CD8^+^ TILs, for example, migrate from the peripheral blood into the TME and can directly kill tumor cells through the release of perforin and granzymes, leading to apoptosis of target cells [120]. Notably, the density and localization of tumor-infiltrating cytotoxic CD8^+^ T cells, especially within cancer cell nests, are strongly associated with improved prognosis and survival in multiple solid tumors, including breast cancer [121], non-small cell lung cancer [122], and colorectal cancer [123].

Chronic exposure to tumor antigens and sustained T cell receptor signaling in the tumor microenvironment drives a progressive, dysfunctional exhaustion-like differentiation program in CD8⁺ TILs, with a reduction in canonical effector functions such as proliferation, cytokine production, and cytotoxicity, and the upregulation of multiple inhibitory receptors (e.g., PD-1 and LAG-3) and the acquisition of distinct transcriptional and epigenetic signatures. Within the tumor, exhausted T cells are also heterogeneous, encompassing stem-like/precursor subsets that retain some responsiveness and the capacity to proliferate upon checkpoint blockade, as well as more terminally exhausted cells with limited functional plasticity. The exhausted state represents a major barrier to effective antitumor immunity and underlies, at least in part, the variable efficacy of immunotherapies such as PD-1/PD-L1 blockade, which can reinvigorate certain exhausted T cell subsets but often fails to fully restore antitumor activity in solid tumors.

In addition to classical T cell exhaustion, CD8⁺ TILs can enter an anergy-like dysfunctional state when they encounter tumor antigens in a prolonged fashion in the absence of costimulation, such as CD28 engagement or adequate inflammatory signals, and often with insufficient or altered CD4⁺ T cell help [124]. In this context, antigen recognition without appropriate costimulatory cues leads to a hyporesponsive program characterized by diminished effector cytokine production and impaired proliferation, reminiscent of peripheral T cell anergy observed in tolerance models, and distinct from the later exhaustion phenotype driven by chronic antigen stimulation and inhibitory receptor upregulation. This anergy-like dysfunction contributes to ineffective antitumor immunity through the establishment of a state in which tumor-reactive T cells are present but unable to induce robust cytotoxic responses.

Tumor-resident memory cells (T_RM_) represent an additional important population of TILs that persist within tumor tissue, where they play a central role in local antitumor immunity. T_RM_ cells are typically characterized by the expression of markers such as CD69 and CD103, low or absent expression of egress receptors such as S1PR1 and CCR7, and transcriptional programs that promote tissue retention [125]. Functionally, T_RM_ cells are often enriched in tumor-reactive clones, exhibit rapid effector responses, and have potent cytotoxic effects, allowing them to rapidly respond to malignant cells within the tumor microenvironment. Accumulating evidence indicates that TGF-β is essential for the formation and maintenance of T_RM_ cells in tumors through the induction of CD103 expression on activated CD8^+^ T cells and for the regulation of T_RM_ cell effector functions through bidirectional integrin signaling [126, 127]. Notably, excessive levels of TGF-β are capable of limiting T cell infiltration in tumors and promoting exhaustion, highlighting the dual role of this cytokine [128–130]. Notably, T_RM_ cells exhibit unique metabolic adaptations that support survival in hypoxic and nutrient-poor environments. First, in T_RM_ cells, glycolysis increases under hypoxic conditions because of the upregulation of glucose transporters and glycolytic enzymes, which compensate for impaired oxidative phosphorylation. This shift allows them to perform effector functions, such as those of granzyme B and IFNγ, even when oxygen is limited [131]. Second, T_RM_ cells depend on nonsteroidal products of the mevalonate-cholesterol pathway, particularly coenzyme Q, which is driven by increased SREBP2 activity. Increased coenzyme Q synthesis promotes mitochondrial respiration and supports the formation of memory T cells and antitumor immunity in nutrient-restricted environments [132]. Third, T_RM_ cells demonstrate metabolic flexibility by utilizing alternative carbon sources and maintaining mitochondrial fitness. For example, they can divert succinate out of the tricarboxylic acid (TCA) cycle for autocrine signaling via SUCNR1 and depend on pyruvate carboxylase–mediated anaplerosis to replenish TCA cycle intermediates. This is crucial when glucose and oxygen are scarce [133]. These characteristics enable effective immune surveillance and tumor control by T_RM_ cells [134]. Accordingly, high densities of T_RM_ cells in tumor tissue correlate with longer survival and better responses to immunotherapy across multiple cancer types [135]. Strategies to enhance T_RM_ cell formation through vaccines, adjuvants, or intratumoral cytokine therapy are therefore being investigated to bolster tumor immunity. However, one major challenge for this is the unanswered question about the ontogeny of T_RM_ cells, with evidence suggesting that they originate from circulating precursors, such as T_PEX_ cells, or differentiate them from effector or memory-like cells within the TME [136, 137].

In addition to this plethora of tumor-reactive TILs, CD8^+^ TILs that are activated independent of tumor antigens, known as bystander TILs, are involved in the antitumor T cell response. In diverse tumor types, bystander TILs bind peptides from various viruses, including cytomegalovirus (CMV), Epstein–Barr virus (EBV), and influenza A [138, 139]. Bystander TILs infiltrate tumors as part of the immune response, but they generally recognize cancer cells in a TCR-independent manner. Following activation by the proinflammatory cytokines IL-15, IL-2, and IL-12 or distinct metabolites, they increase the expression of cytotoxic molecules, such as NKG2D, and produce effector cytokines, particularly IFNγ and TNF, thereby enhancing antitumor immune capacity [140–143]. Notably, they phenotypically differ from tumor-reactive CD8^+^ TILs, e.g., by a lack of CD39 expression [144]. Hence, there is high heterogeneity within the CD8^+^ TIL population characterized by different adaptation mechanisms, reflecting the complex and dynamic immune/tumor interplay.

### Immune evasion mechanisms of the TME directed against CD8^+^ TILs

The TME is the complex and dynamic ecosystem surrounding and infiltrating a tumor. This ecosystem is composed of cancer cells, as well as a diverse array of nonmalignant cellular and acellular components. The TME includes different types of immune cells, cancer-associated fibroblasts, endothelial cells, pericytes, and other tissue-resident cell types [145, 146]. It is therefore not surprising that the TME profoundly shapes the fate and function of CD8⁺ TILs beyond providing antigenic signals. In particular, antigenic, cellular, structural, and metabolic signals are integrated. For example, immunosuppressive cells, including regulatory T cells, MDSCs, and tumor-associated macrophages (TAMs), limit CD8⁺ TIL activity. MDSCs and TAMs both produce the inhibitory cytokines IL-10, IL-6, and TGF-β [147, 148]. MDSCs also express the chemokines CCL3, CCL4, and CCL5, resulting in an inhibitory orchestration of the antitumor immune response, including the induction of immunosuppressive regulatory T cells and the suppression of CD8^+^ TIL and natural killer cell activity in tumors [147]. In addition to producing IL-10, TGF-β, and IL-6, TAMs produce VEGF and CCL2, promoting immunosuppression, angiogenesis, tissue remodeling, and tumor growth and metastasis [148]. In addition to inhibitory cytokines and chemokines, immunosuppressive cells act through metabolic competition and express immune checkpoint ligands for inhibitory receptors, such as PD-L1 ligands. PD-L1 upregulation on immune and tumor cells is induced by IFNγ and subsequent signaling via the JAK1/JAK2-STAT1/STAT2/STAT3-IRF1 axis. IRF1 then binds directly to the PD-L1 promoter [149]. PD-L1 binding to PD-1 dampens TCR signaling, reducing proliferation, cytokine production, and cytotoxic function [150]. However, PD-L1 is not the only inhibitory ligand whose expression is upregulated. CD80/CD86 (CTLA-4 and CD28 ligands) engage CTLA-4 to outcompete the costimulatory receptor CD28 and limit T cell activation. Moreover, the TIM-3 ligand galectin-9, which is induced by interferons, regulates T cell death and inhibits TIL function [151]. Similarly, both CD155 (the ligand for TIGIT) and HVEM (the ligand for BTLA) participate in the coinhibitory receptor module that drives immune suppression and tumor immune escape by inhibiting TIL and NK cell function [152, 153].

The TME is also metabolically hostile, with hypoxia, nutrient depletion, and the accumulation of suppressive metabolites impairing T cell survival and effector function. Tumor and stromal cells compete with effector immune cells for essential nutrients, notably glucose, glutamine, and amino acids such as tryptophan. This competition results in the local depletion of these substrates, which impairs T cell proliferation and effector function [154, 155]. In addition, hypoxia, a key feature of the TME, stabilizes hypoxia-inducible factors (HIFs). HIF subunit 1α (HIF-1α) further impairs immune cell function by increasing PD-L1 expression [156]. HIFs further stimulate the production of indoleamine 2,3-dioxygenase (IDO) and other immunosuppressive molecules [157, 158]. IDO catalyzes the degradation of tryptophan into kynurenine, which results in tryptophan starvation and the accumulation of immunosuppressive metabolites. This process directly inhibits the proliferation of effector T cells and promotes the differentiation of regulatory T cells, contributing to immune escape [159]. Hypoxia also affects antigen presentation and promotes TAP and likely ERAAP deficiency, resulting in the downregulation of MHC class I and its immunopeptidome [160–162]. Furthermore, metabolic reprogramming of tumor cells results in increased production of lactate by aerobic glycolysis. High lactate levels suppress CD8⁺ TIL function by acidifying the microenvironment; inhibiting proliferation, cytokine production, and cytotoxicity; and disrupting T cell metabolism, thereby promoting dysfunction and limiting antitumor immunity and representing a metabolic checkpoint in the TME [163]. Lipid accumulation, which is associated with CD36-mediated TIL dysfunction, is also commonly observed in the metabolically reprogrammed TME [164, 165]. CD36 is expressed on various cell types within the TME, including CD8^+^ TILs. It is involved in lipid metabolism-mediated angiogenesis, inflammatory responses, antigen presentation, and regulatory T cell survival and function [166–169]. Together, these mechanisms promote a metabolically unfavorable and immunosuppressive environment that reduces the efficacy of antitumor immune responses and resistance to immunotherapies [170].

Spatial organization within tumors further influences CD8⁺ TIL behavior, with distinct niches supporting stem/precursor-like, exhausted, or tissue-resident memory-like states, and is reflected by different so-called immunotypes identified and defined in several solid tumor types. For example, the degree and composition of T cell infiltration vary widely among different tumor types. This has been recently shown in a large cohort of 6021 primary tumors from 43 different carcinoma entities [171]. A highly inflamed pancancer cluster had the highest density of CD8^+^ TILs and the strongest interactions between these cells and CD4^+^ T helper cells, dendritic cells (DCs), and M2 macrophages. This cluster is strongly associated with a favorable tumor stage, supporting the idea that juxtaepithelial cytotoxic TILs play a key role in the inflamed tumor immune phenotype [171]. However, not every cancer entity exhibits the same degree of T cell infiltration as its most extreme form of T cell exclusion induced by physical barriers and stromal remodeling. This process is characterized by a dense extracellular matrix (ECM) produced by cancer-associated fibroblasts (CAFs), leading to increased matrix stiffness and altered tissue architecture [172]. The activation of CAFs and the subsequent induction of ECM production can be mediated by TGF-β [128]. Additionally, a disorganized vascular network driven mainly by VEGF and increased interstitial fluid pressure hinders T cell trafficking into tumors [173, 174]. Pancreatic cancer is an example of a tumor with a deficiency in immune cell infiltration resulting from the integration of multiple immunosuppressive mechanisms. More precisely, the TME of pancreatic cancer is characterized by an “immune desert”, which is characterized by low immune cell infiltration, dense fibrotic stroma formation, and a profoundly immunosuppressive metabolic landscape [175]. Compared with other tumors, pancreatic cancer has a low number of somatic mutations, and this low tumor mutational burden (TMB) results in a limited generation of tumor neoantigens that can be recognized by immune cells [176, 177]. In addition, the immunosuppressive environment compromises the maturation and activation of dendritic cells, causing defective antigen presentation. Along these lines, pancreatic cancer is characterized by reduced numbers of TILs and, in particular, reduced numbers of cytotoxic CD8^+^ effector T cells [178]. The immunosuppressive milieu of the TME is further characterized by increased activity of Tregs, which hinder the proliferation and activation of effector T cells via the secretion of immunosuppressive cytokines, such as IL-10 and TGF-β [179]. Additionally, the few CD8^+^ TILs predominantly exhibit functional exhaustion with high expression of inhibitory receptors, including PD-1, TIM-3, and LAG-3 [180]. Together, these features of the TME constrain antitumor immunity and determine CD8^+^ TIL adaptation and fate while also creating selective vulnerabilities that need to be therapeutically targeted to enhance CD8⁺ TIL function.

### Targeting chronically adapted CD8^+^ TILs

On the basis of this understanding of immune/tumor interplay for cancer development and progression, immunotherapy has evolved as a promising alternative for cancer treatment. In contrast to previous treatment strategies targeting oncogenic signaling, immunotherapy modulates and utilizes the patient’s own immune system to target cancer cells. Additionally, the first data reveal that the combination of both interventions, interference with oncogenic signaling and immunotherapy, may represent an even more effective measure. These findings underscore that the highly adaptive circuit of tumors and the immune system must be disrupted for effective cancer therapy, with CD8^+^ TILs serving as a critical effector component.

#### Immune checkpoint blockade (ICB)

One promising strategy to harness the immune system to fight cancer is ICB therapy, which relieves inhibitory signals that, among others, restrain TIL activity. The most clinically relevant targets are the PD-1/PD-L1 and CTLA-4 checkpoint pathways, which restore antitumor immune responses [9, 181]. ICB therapy has transformed the treatment landscape for multiple malignancies mediated by immune checkpoint inhibitors that are monoclonal antibodies targeting immune checkpoint receptors or ligands [182, 183]. ICB affects key aspects of T cell receptor and CD28 costimulatory signaling, leading to increased proliferation, cytokine production, metabolic fitness, and cytotoxic activity in CD8⁺ TILs, including chronically adapted exhausted TILs, as well as infiltration into the tumor microenvironment [184]. Rather than fully reprogramming dysfunctional TILs, checkpoint blockade primarily acts on a subset of TCF-1^+^PD-1^+^ stem-like exhausted CD8^+^ TILs, expanding this pool and enabling their differentiation into more effective T cells [185]. A stable reservoir of TCF-1^+^PD-1^+^ stem-like CD8^+^ TILs has been observed in the tumor-draining lymph nodes (TDLNs) of some tumor types. These T cells can be developmental precursors of TCF-1^+^ intratumoral T cells that are maintained by continuous migration [186, 187] and are primarily responsible for the proliferative burst observed during anti-PD-1 checkpoint immunotherapy because of their high proliferative potential and self-renewal capacity [188]. Consistently, several studies have shown that blocking T cell egress from the TDLN, e.g., by surgically removing the TDLN, impairs the ICB response [189, 190]. Thus, immune checkpoint blockade shifts the balance within the CD8⁺ TIL compartment toward functional antitumor responses by releasing inhibitory constraints imposed by the tumor microenvironment.

Although ICB has revolutionized cancer care, it is effective only in a minority of patients. This lack of responsiveness is multifactorial and involves tumor-intrinsic and tumor-extrinsic mechanisms. Intrinsic tumor factors include genetic and epigenetic alterations that hinder antigen presentation. Examples include the loss of β2-microglobulin or MHC class I molecules [191, 192]. Other factors include mutations in interferon-γ signaling pathways, such as JAK1/2 [193], and the activation of oncogenic pathways, such as the Wnt/β-catenin pathway, the epidermal growth factor receptor (EGFR) pathway, and the loss of PTEN [194]. These alterations suppress immune infiltration and upregulate immunosuppressive molecules. Tumor-extrinsic factors influence the composition and function of the TME. These factors include low infiltration of cytotoxic CD8^+^ T cells, the presence of immunosuppressive cells, and the production of inhibitory metabolites and cytokines that broadly suppress T cell activity [195, 196]. Additionally, the expression of alternative immune checkpoints such as TIM-3, LAG-3, and TIGIT can mediate resistance when the PD-1/PD-L1 or CTLA-4 pathway is blocked [194]. Melanoma patients, for example, have improved cancer outcomes when LAG-3 is blocked in combination with PD-1 blockade [197].

In addition to the combined blockade of several immune checkpoints, several experimental and preclinical approaches involving combination therapies, including targeted metabolic programming, cytokines, vaccines, and epigenetic modulators, have been proposed. Targeted metabolic programming enhances the efficacy of ICIs and involves lipid, glucose, amino acid, and adenosine metabolism. These pathways increase the number of CD8^+^ TILs more effectively than ICIs alone do [198]. For example, a correlation between checkpoint resistance and a hypermetabolic tumor phenotype with upregulation of the glycolytic, oxidoreductase, and mitochondrial oxidative phosphorylation pathways in melanoma patients has been described [199]. Consequently, the glycolytic potential, effector function, and ability to expand of tumor-specific TILs decrease in response to ICB. Therefore, targeting glucose uptake and glucose-metabolizing enzymes could be a potential additive target in ICB to enhance ICI immunotherapy. One approach might be LDH inhibition, which has been shown to decrease glucose uptake by tumor cells and the expression of glucose transporter 1 (GLUT1) while increasing glucose uptake and GLUT1 expression by tumor-infiltrating T cells [200]. Another approach involves the direct inhibition of GLUT1, which is overexpressed in many human cancers [201]. Although metabolic reprogramming may enhance the efficacy of ICB in different cancer types, further studies are needed to determine the clinical benefits of these interventions.

Several studies have investigated the effects of combination therapy with ICIs targeting the PD-1/PD-L1 axis and cytokine-based immunomodulators designed to enhance T cell activation and proliferation. For example, in murine colon carcinoma models, ICIs synergize with CD25-biased anti-IL-2 antibodies, which selectively stimulate the expansion and effector functions of tumor-specific CD8^+^ T cells in a CD25-dependent manner, overcoming Treg-mediated suppression [202]. Like IL-2, IL-15 stimulates the proliferation and effector functions of CD8^+^ TILs [203]. Indeed, when combined with ICB, the use of the IL-15 superagonist receptor linker significantly increases the release of IFNγ by TILs in patients with renal cell adenocarcinoma, suggesting a beneficial role for the combination therapy [204]. Similarly, the combination of ICB with agonists of costimulatory molecules such as OX40, 4-1BB, and CD27 also shows promise for increasing effector T cell responses and redirecting TIL dysfunction.

Moreover, the combination of ICB with vaccines or antiviral therapies could also positively influence the quantity and functionality of tumor-specific CD8^+^ T cells. For example, cancer vaccines aim to prime and/or expand tumor-specific T cell responses by presenting tumor antigens in an immunogenic context, often promoting the activation of naive or memory T cells and guiding them toward effector or tissue-resident states [205]. Combining ICB with cancer vaccines can be synergistic, increasing the pool of tumor-reactive T cells (by the vaccine) and simultaneously sustaining and amplifying their function (by ICB) within the immunosuppressive tumor milieu. Ongoing research focuses on optimizing antigen selection, delivery platforms, and combination timing to maximize clinical benefit.

Recently, it has been shown that SARS‑CoV‑2 mRNA vaccines can increase the efficacy of ICB in cancer [206]. Patients vaccinated within 100 days of ICB initiation exhibited improved survival, including those with tumors typically considered cold or poorly responsive to immunotherapy. Preclinical studies suggest that the vaccine triggers type I interferon responses, promotes immune activation and epitope spreading, and increases tumor PD‑L1 expression, collectively sensitizing tumors to ICB [61]. These findings raise the possibility that widely available “off‑the‑shelf” mRNA vaccines could be repurposed to increase immune checkpoint blockade (ICB) responses and improve cancer patient outcomes, but these findings require further clinical validation.

A further approach for improving the response rates of ICB is the combination with epigenetic modulators, which are able to increase ICB efficacy by increasing tumor immunogenicity, modulating the immune microenvironment, and reversing resistance mechanisms. Epidrugs include inhibitors of DNA methyltransferase (DNMT), histone deacetylase (HDAC), bromodomain (BET), or histone demethylases (HDMT). Many combination therapies involving epidrugs and ICIs for solid tumors and hematologic malignancies are currently being tested in clinical trials (see review in [207]). Regardless of the combined approach for supplementing ICB, ongoing and future trials are necessary to evaluate the safety and effectiveness of next-generation ICB regimens.

#### Adoptive cell therapies

**Fig. 4:**
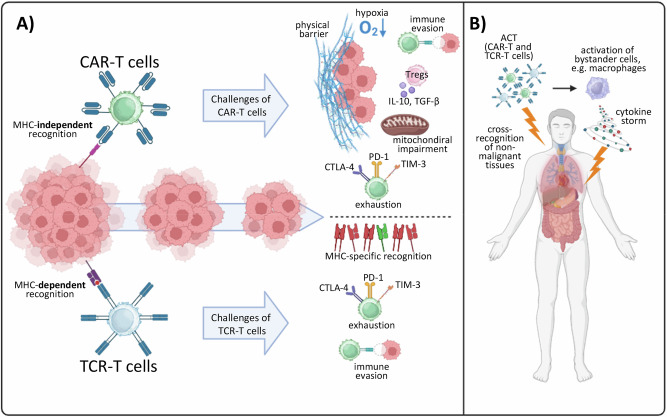
ACT in cancer therapy. **A** Mechanisms of action and current challenges of CAR-T cells and TCR-T cells. **B** Adverse effects of ACT include cross-recognition of nonmalignant tissues and direct tissue damage via cytokines. ACT adoptive cell therapy, CAR-T chimeric antigen receptor-T cell, TCR-T T cell receptor-T cell, TCR T cell receptor, MHC major histocompatibility complex

In adoptive cell therapy (ACT), immune cells are isolated, modified, and expanded before reinfusion into the patient to increase antitumor immune responses (Fig. 4) [208]. Therefore, this therapeutic strategy can change the makeup of TILs. Chimeric antigen receptor (CAR) T cell therapy is a type of ACT. CAR-T cells are autologous or allogeneic T lymphocytes that have been genetically engineered to express synthetic receptors. These receptors allow cells to recognize and kill cells that express specific antigens, which are most commonly found on malignant cells, independent of MHC presentation [209, 210]. CAR-T cell therapy is limited by low efficacy because of tumor immune evasion and chronic adaptation mechanisms. These mechanisms include antigen heterogeneity, exhaustion-like dysfunction, an immunosuppressive and hypoxic TME, and physical barriers that prevent CAR-T cells from infiltrating the tumor [211, 212]. Interestingly, compared with unselected T cells, CAR-T cells derived from defined CD8^+^ and CD4^+^ subsets exhibit more consistent and potent antitumor activity, and combining both subsets can result in synergistic effects [213]. Combining CAR-T cell therapy with ICIs has been shown to prevent CAR-T cell exhaustion [214]. Moreover, overexpression of the AP-1 factor c-Jun is associated with resistance to exhaustion, suggesting that engineering “next-generation” CAR-T cells in a manner in which they overexpress c-Jun might be a promising approach to render them resistant to exhaustion-like chronic adaptation [215].

T cell receptor-T cell (TCR-T) therapy involves the use of autologous or allogeneic T cells that have been genetically engineered to express a specific T cell receptor (TCR) that recognizes a tumor-associated antigen. An important difference from CAR-T cells is that they are able to target intracellular antigens [216]. However, unlike CAR-T cells, which recognize cancer cells independently of MHC presentation, TCR-T cells recognize only peptide antigens when they are presented with specific HLA molecules [217]. Consequently, TCR-T cells are effective only in patients who express matching HLA alleles, limiting the eligible patient population and necessitating HLA typing prior to therapy [218]. Tumor cells are also capable of downregulating or losing HLA expression. This impairs antigen presentation, enables immune evasion, and reduces the effectiveness of TCR-T cell therapy. Resistance to TCR-T cell therapy can also occur through antigen escape. The loss or mutation of the target antigen in tumor cells promotes a lack of recognition of the antigen by TCR-T cells [219]. In addition, the effector function of TCR-T cells progressively decreases, accompanied by the expression of inhibitory receptors and altered transcriptional and epigenetic states characteristic of exhaustion, thereby limiting the antitumor effect of TCR-T cell therapy [220]. Interestingly, targeting the transcriptional regulators TOX or NR4A1, either genetically or pharmacologically, has been shown to counteract this exhaustion-like dysfunction of TCR-T cells, but its clinical feasibility and effectiveness remain unclear [221–223].

#### Adverse effects caused by interference with tumor/immune adaptation

Interference with the tightly adapted tumor/immune interplay comes with costs. ICB and ACT can induce immune-related adverse events because of heightened immune activation (Fig. 4). ICB commonly triggers autoimmune-like toxicity affecting the skin, gastrointestinal tract, liver, endocrine organs, and lungs, including rash, colitis, hepatitis, thyroiditis, and pneumonitis, reflecting off-target T cell activation in healthy tissues. ACTs, including CAR-T cells or TCR-engineered T cells, are often associated with cytokine release syndrome (CRS) and immune effector cell–associated neurotoxicity syndrome (ICANS), which can range from mild fever and fatigue to severe systemic inflammation and neurological symptoms [224–226]. On the one hand, these events occur when engineered T cell receptors (TCRs) or chimeric antigen receptors (CARs) recognize antigens resembling the intended target that are present on nonmalignant cells [227–229]. This cross-reactivity has resulted in severe and sometimes fatal toxicity in clinical trials. On the other hand, a systemic hyperinflammatory response is triggered by the activation of bystander immune cells, especially macrophages, which amplify cytokine production, resulting in markedly elevated circulating levels of proinflammatory cytokines, such as IL-6, TNF, and IFNγ [230]. Both therapies can also lead to hematologic, cardiovascular, or infectious complications due to immunosuppression or tissue damage. Careful monitoring, early intervention, and immunosuppressive management are critical to mitigate these potentially severe toxicities while maintaining antitumor efficacy.

## Conclusion and future perspectives

The adaptation of T cell immunity to chronic antigen exposure is a central barrier to the control of chronic infections and tumors. Once regarded as a dysfunctional or failing state of T_EX_ cells, chronically adapted T cells are now recognized as a distinct type of differentiation shaped by persistent antigen exposure and microenvironmental constraints. Their heterogeneity, especially the presence of a stem-like/precursor subset, presents both a challenge and a therapeutic opportunity. Immune checkpoint blockade has demonstrated the ability to increase the number of chronically adapted T cells; however, the current limits of this approach highlight the need for deeper mechanistic insights and novel strategies. Future therapeutic strategies require a strong focus on intervening with the heterogeneous adaptive programs of T cells to sustain durable antitumor or antiviral immunity while minimizing adverse effects. Rather than viewing therapy solely as the reinvigoration of dysfunctional T cells, emerging approaches aim to prevent or redirect maladaptive differentiation by modulating antigen exposure, costimulatory signaling, metabolic constraints, and epigenetic fixation within the tumor microenvironment of infected tissues. In this context, spatial immune architectures are poised to serve as informative biomarkers, as the localization, organization, and cellular interactions of tissue/tumor-infiltrating T cells provide critical insight into functional states and therapeutic responsiveness. Integrating these spatial, temporal, and clonal insights with combinatorial strategies, such as ICB, vaccines, metabolic reprogramming, cytokine support, modulation of oncogenic signaling, or targeted manipulation of tissue-resident and stem-like T cell subsets, holds promise for achieving more precise, durable, and effective immunotherapies in chronic infection and cancer.
